# Composition and crystalline properties of TiNi thin films prepared by pulsed laser deposition under vacuum and in ambient Ar gas

**DOI:** 10.1186/1556-276X-7-37

**Published:** 2012-01-05

**Authors:** Jeong Ok Cha, Tae Hyun Nam, Mohammad Alghusun, Jeung Sun Ahn

**Affiliations:** 1 Department of Physics, Kyung Hee University, Seoul, 130-701, South Korea; 2 School of Nano and Advanced Materials Engineering, Gyeong Sang National University, Gyeongnam, 660-701, South Korea

## Abstract

TiNi shape memory alloy thin films were deposited using the pulsed laser deposition under vacuum and in an ambient Ar gas. Our main purpose is to investigate the influences of ambient Ar gas on the composition and the crystallization temperature of TiNi thin films. The deposited films were characterized by energy-dispersive X-ray spectrometry, a surface profiler, and X-ray diffraction at room temperature. In the case of TiNi thin films deposited in an ambient Ar gas, the compositions of the films were found to be very close to the composition of target when the substrate was placed at the shock front. The *in-situ *crystallization temperature (*ca*. 400°C) of the TiNi film prepared at the shock front in an ambient Ar gas was found to be lowered by *ca*. 100°C in comparison with that of a TiNi film prepared under vacuum.

## Background

In recent years, shape memory materials have attracted much attention as functional materials owing to a variety of industrial and medical applications. The shape memory effect and superelasticity, especially in smart materials [1] and microelectromechanical systems [MEMS], have been extensively investigated in the past decades due to their potential use in several applications. TiNi shape memory alloys [SMAs] are most suitable for MEMS and Bio-MEMS microactuators (e.g., micropump [2,3], microwrapper [4], and blood vessels [5]) because of their large working distance compared to bimetals and piezo materials. However, slow thermal response due to a low cooling rate in bulk SMA is a barrier for MEMS application. On the other hand, TiNi thin films has only a small amount of thermal mass to heat or cool, thereby substantially minimizing the response time and enhancing the speed of operation.

Several techniques have been used for the deposition of TiNi SMA thin films, such as sputtering [2,6-10], pulsed laser deposition [11-15], flash evaporation [16], and cathodic arc plasma ion plating [17]. One of the major problems in fabricating TiNi thin films is the composition control. The composition control is essential to adjust the working temperature of the microdevice as the composition ratio strongly influences the transformation temperature of the TiNi SMA [18]. Another major obstacle related to the growth of TiNi thin films is the crystallization of TiNi thin films since only crystalline TiNi thin films have the shape memory effect. Most researchers have fabricated TiNi thin films using conventional sputtering methods, wherein it is difficult to control the composition of TiNi thin films [19]. Moreover, TiNi thin films fabricated by sputtering at ambient temperature are often amorphous and thus require a post-annealing process to obtain a shape memory effect [2,20].

Pulsed laser deposition [PLD] method has several advantages over the conventional sputtering methods, such as the following: (1) it preserves the target composition to the substrate stoichiometrically [11], and (2) it is flexible to use either in high vacuum or in an ambient gas. Koji et al. was the first to report the TiNi shape memory alloy thin films deposited using PLD in vacuum, although the growth rate (2.4 × 10^-4 ^nm per pulse) of the thin film was too low for a microactuator [10]. To fabricate smooth TiNi SMA thin films with sufficient thickness in reasonable deposition times, Gu et al. investigated the optimum deposition parameters (i.e., the target-substrate distance and the rotation speed of the target) in PLD under vacuum [11]. Lu et al. studied the influence of substrate temperature on the properties of the TiNi films deposited using PLD under vacuum [12]. The substrate temperature, target-substrate distance, etc. are known to play an important role in the composition control and the crystallization of the films in the PLD method under vacuum.

In the present paper, TiNi thin films using the equiatomic TiNi target under vacuum and in an ambient Ar gas were deposited by PLD and studied. The influence of Ar atmosphere on the composition, thickness, and *in-situ *crystallization temperature of TiNi thin films is observed.

## Methods

The PLD system for the deposition of TiNi thin fills was utilized in this work. The equiatomic TiNi target is placed in a holder rotating at 20 rpm and irradiated by a focused KrF excimer laser (COMPex 102, Lambda Physik AG, Goettingen, Germany) at 45° with an energy density of 1.24 J/cm^2 ^and a repetition rate of 16 Hz. The PLD time was 1 h. Before deposition, all targets were polished with grade 1200 SiC metallographic papers to minimize the formation of large particles on the surface of the thin films and then cleaned with methanol in an ultrasonic cleaner to minimize contamination before deposition. The substrates, Si (100), are glued by a silver paste to a heater located in front of the target at a distance of 25 to 50 mm.

In order to investigate the influences of an ambient gas on the composition and the thickness of TiNi thin films, the films were placed under high vacuum (5 × 10^-6 ^Torr) and in a 200-mTorr Ar gas. In both cases, the base pressure before deposition was 2 × 10^-6 ^Torr. The TiNi thin films were deposited on Si (100) substrates at various substrate temperatures raging from room temperature to 600°C. After deposition, the composition of the films was measured by energy-dispersive X-ray spectrometry [EDXS] (EMAX-5770, HORIBA Ltd., Minami-Ku, Kyoto, Japan). The thickness of the deposited films was measured with a surface profiler (DEKTAK 3030, Veeco Instruments Inc., Plainview, NY, USA). X-ray diffraction [XRD] technique was employed to study the crystal structures of the films deposited at various substrate temperatures.

## Results and discussion

**Figure 1:**
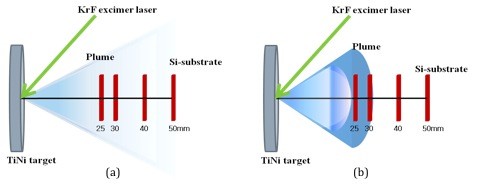
**Schematic diagram of the shape of plumes**. The plumes were ablated under (**a**) high vacuum (5 × 10^-6 ^Torr) and in (**b**) ambient Ar gas of 200 mTorr.

The shape of plumes generated under high vacuum (5 × 10^-6 ^Torr) and in 200-mTorr Ar gas is schematically illustrated in Figure 1. In the case of PLD under vacuum deposition as shown in Figure 1a, no clear boundary of the plume is observed by the naked eye as the fluorescence of the plume is weak. On the other hand, in the case of PLD in ambient Ar gas, Figure 1b, a conical plume with a spherical tip is noticed, and the dense fluorescence from all parts of the plume and shock front [21] at the front of the plume are observed.

**Figure 2:**
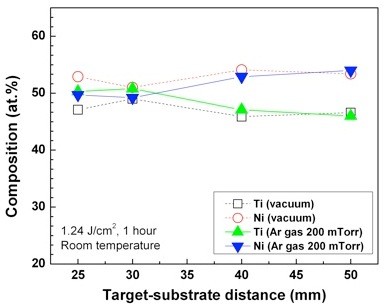
**The compositions of TiNi thin films deposited on Si substrate at room temperature**.

**Figure 3:**
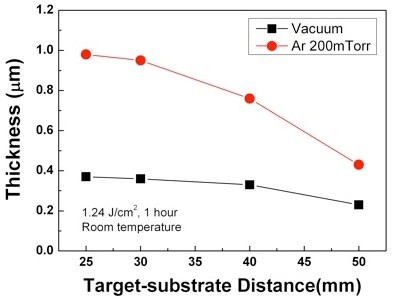
**Thickness of TiNi thin films deposited on Si substrate at room temperature for various target-substrate distances**.

In order to investigate the influence of ambient Ar gas on the composition and thickness of TiNi thin films, TiNi thin films under vacuum and in 200-mTorr Ar atmosphere at various target-substrate distances were prepared. Figure 2 shows the composition variation of the TiNi thin films deposited under vacuum and in 200-mTorr Ar atmosphere as a function of the target-substrate distance. The composition of the thin films deposited under vacuum significantly differs from the target's composition, except for a distance of 30 mm. On the other hand, the compositions of the thin films deposited in 200-mTorr Ar atmosphere are found to be very close to the target's composition when the substrate is placed inside of the plume and to deviate from the target's composition when the substrate is placed outside of the plume. Figure 3 shows the thickness of the TiNi thin films deposited under vacuum and in 200-mTorr Ar atmosphere. The thicknesses of the thin films deposited in 200-mTorr Ar atmosphere are found to be thicker than those under vacuum over the entire target-substrate distance range. In addition, the growth rate largely increases when the substrate is placed within the plume for the deposition in Ar gas atmosphere. Results of Figures 2 and 3 clearly indicate that the 200-mTorr Ar atmosphere plays an important role on the composition control and enhanced thickness of the TiNi thin films deposited using the PLD method.

When PLD is performed in a low-pressure ambient atmosphere or under vacuum, ablated species simply move swiftly in a forward-directed distribution. On the other hand, in a high-pressure ambient atmosphere, ablated species jostle with ambient molecules, and the flight velocity gradually decreases due to collisions between the ablated species and ambient molecules, and finally, the ablated species cease to move. Therefore, a significant number of ablated species and ambient molecules are accumulated, resulting in the formation of high-density ablated species and high-density ambient molecules in the impedance region. This region is called the shock front [21-24].

The enhanced thickness in Ar atmosphere at the entire target-substrate distance range than that under vacuum is attributed to the collisions between the ablated Ti/Ni species and the Ar ambient molecules. Because of the collisions, more Ti and Ni species can reach a substrate in Ar atmosphere as compared to those under vacuum deposition over the entire target-substrate distance range. Moreover, the greatly enhanced thicknesses of TiNi thin films at distances of 25 mm and 30 mm in the case of the 200-mTorr Ar atmosphere are caused by the shock front consisting of high-density ablated Ti and Ni species. In this paper, a 200-mTorr Ar pressure is high enough to form the shock front, and distances of 25 mm and 30 mm may be a region of the shock front. Also, the result of Figure 2 could be explained by the shock front. A number of collisions between the ablated species(Ti/Ni) and Ar gas molecules are increased at the shock front. Therefore, the ablated species(Ti/Ni) move at a constant velocity, despite of the different masses of Ti and Ni. Because of this, the compositions of the thin films are very close to the target's composition when the substrate is placed at the shock front. Compositions of the thin films deposited using conventional sputtering method were greatly different from the target's composition, necessitating the need of suitable methods to control the composition. The result presented here implies that TiNi thin films which are close to the target's composition using PLD at the shock front in 200-mTorr Ar atmosphere are obtained easily.

**Figure 4:**
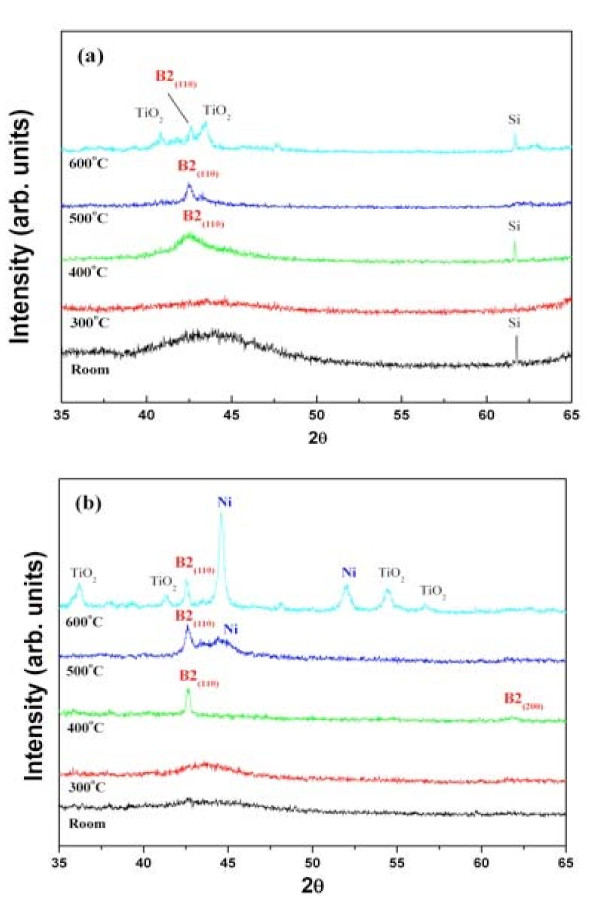
**XRD patterns of TiNi thin films deposited on Si substrate at various substrate temperatures**. In (**a**) high vacuum and (**b**) 200-mTorr Ar atmosphere.

The crystalline TiNi thin films with shape memory effect are required for MEMS applications. To obtain *in-situ *crystalline TiNi thin films, TiNi films were deposited on the Si(100) substrate at high temperatures. In the case of the sputtering method, it is reported that TiNi thin films were crystallized at a substrate temperature of 550°C [25]. The target-substrate distance was fixed at 30 mm to realize both a high deposition rate and low density of droplets on the film surface. In order to investigate the influence of the shock front on the *in-situ *crystallization of the TiNi thin films, TiNi thin films on Si substrates at various temperatures ranging from room temperature to 600°C were deposited using PLD in an Ar atmosphere and under high vacuum. Figure 4a, b shows the XRD patterns of TiNi thin films measured at room temperature on the Si (100) substrate. Figure 4a shows the XRD pattern of TiNi thin films deposited under high vacuum (5 × 10^-6 ^Torr) at various substrate temperatures. The XRD pattern of TiNi thin films deposited under vacuum at above 500°C of substrate temperature shows a diffraction peak at 2*θ *≈ 42.7°, corresponding to the major principal reflection (110) of the austenitic B2 phase. However, a distinct TiO_2 _peak can be observed in the XRD pattern of TiNi films deposited on a substrate temperature of 600°C. On the other hand, the XRD pattern of TiNi thin films deposited in 200-mTorr Ar atmosphere shows a major diffraction peak at 2*θ *≈ 42.7° when the substrate temperate is at 400°C or higher. In addition to this diffraction peak, a minor diffraction peak at 2*θ *≈ 61.2°, corresponding to the reflection (200) of the B2 phase, is also observed in the XRD pattern of the TiNi film deposited at 400°C in 200-mTorr Ar atmosphere. When the substrate temperature is raised up to 600°C, distinct TiO_2 _and Ni peaks can be observed in the XRD pattern. The reason that the pure Ni peak increased in intensity is that Ti reacts with oxygen, producing Ti_2_O so that the rest of Ni cannot combine with Ti. From the XRD analysis of TiNi films deposited at various substrate temperatures, it can be concluded that the shock front plays an important role in lowering the crystallization temperature, *ca*. 100°C, of the TiNi film in 200-mTorr Ar atmosphere in comparison to that prepared under vacuum. The shock front can induce the ablated species (Ti/Ni) which arrive at the substrate slowly as their flight velocity is gradually decreased by the collisions between the ablated species and ambient molecules.

**Figure 5:**
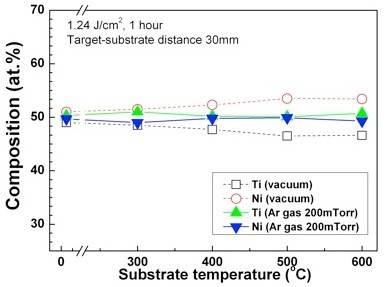
**The composition of TiNi thin films deposited on Si substrate at various substrate temperatures**.

**Figure 6:**
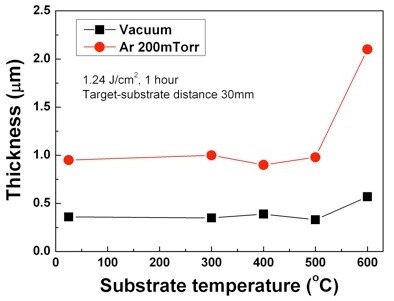
**Thickness of TiNi thin films deposited on Si substrate at various substrate temperatures**.

The influence of substrate temperature on the composition and thickness of the films was investigated, and the results are displayed in Figures 5 and 6. TiNi thin films were deposited at a target-substrate distance of 30 mm in 200-mTorr Ar atmosphere and under vacuum. The substrate temperatures were changed from room temperature to 600°C. From the results of Figure 5, it is found that all the thin films have compositions close to the target composition regardless of the substrate temperature in 200-mTorr Ar atmosphere. In the case of TiNi films deposited under vacuum, the composition of the thin films is influenced by the substrate temperature. Lu et al. [13] reported that substrate temperature influences the composition of the TiNi thin films deposited by PLD under vacuum. Our result in Figure 6 implies that the composition of a TiNi film can be precisely controlled without bothering the substrate temperature using PLD when the TiNi thin films are deposited at the shock front in 200-mTorr Ar atmosphere. Figure 6 shows the thickness of the TiNi films deposited in 200-mTorr Ar atmosphere and under vacuum at various substrate temperatures. The film thickness is found to be almost constant (about 350 nm/1 μm) when the initial substrate temperature is lower than 500°C. However, when the temperature of the substrate becomes 600°C, it increased up to 570 nm and 2.6 μm. The reason of the drastic increase of thickness at 600°C is believed to be due to the fact that Ti particles react with oxygen, producing TiO_2 _as mentioned in the XRD data in Figure 4.

## Conclusions

In order to investigate the influence of ambient Ar gas on the composition, thickness, and *in-situ *crystallization of TiNi thin films, the TiNi films were fabricated by PLD method under vacuum (5 × 10^-6 ^Torr) and in 200-mTorr Ar atmosphere. The deposited films were characterized by EDXS, a surface profiler, and XRD at room temperature. The results are summarized as follows: (1) The thicknesses of the thin films were found to be larger in an Ar gas atmosphere due to the influence of the shock front. (2) In the case of the thin films deposited in an ambient Ar gas, the compositions of the films were found to be very close to the composition of target when the substrate was placed at the shock front. (3) The *in-situ *crystallization temperature (*ca*. 400°C) of the TiNi film prepared at the shock front in an ambient Ar gas was found to be lowered by *ca*. 100°C in comparison with that of a TiNi film prepared under vacuum. (4) The composition of the TiNi thin films deposited on Si substrates at various temperatures from room temperature to 600°C was close to the composition of target, regardless of the substrate temperature. (5) When the temperature of the substrate becomes 600°C, the thickness of the TiNi thin films is largely increased because Ti particles react with oxygen, producing TiO_2_.

## Competing interests

The authors declare that they have no competing interests.

## Authors' contributions

JOC carried out the main part of the experiments and compositional analysis and drafted the manuscript. THN and JSA designed the experimental idea and participated in the structural analysis. All authors read and approved the final version of the manuscript.
